# Supplementary material for: Effects of the Combination of the C1473G Mutation in the Tph2 Gene and Lethal Yellow Mutations in the Raly-Agouti Locus on Behavior, Brain 5-HT and Melanocortin Systems in Mice
Source: Biomolecules. 2023 Jun 8;13(6):963. doi: 10.3390/biom13060963 (PMC10295981; doi:10.3390/biom13060963)
Supplement: Supplementary file 1 [file biomolecules-13-00963-s001.zip › biomolecules-2355616-supplementary.pdf]

Table S1. Hind limbs clasping in young and adult males B6-1473CC and B6-1473GG mice

Trait	B6-1473CC	B6-1473GG	P
		Young (3 week old) mice	
Amount	2.87 ± 1.35, n = 16	13.64 ± 2.28, n = 27	F(1,41)=7.28, p=0.01
Duration, s	14.15 ± 8.72, n = 16	86.89 ± 18.81, n = 27	F(1,41)=7.44, p=0.01
		Adult (12-week-old) mice	
Amount	2.29 ± 0.74, n = 21	4.88 ± 1.07, n = 24	F(1,43)=3.73, p=0.06
Duration, s	14.5 ± 5.23, n = 21	28.4 ± 6.95, n = 24	F(1,43)=2.44, p=0.13
